# Erratum for Wiggins et al., “Inactivating conditions of therapeutic mycobacteriophages”

**DOI:** 10.1128/spectrum.00968-26

**Published:** 2026-07-10

**Authors:** Andrew Wiggins, Umar N. Chaudhry, Fabiana Bisaro, Addison Lueck, Alan A. Schmalstig, Graham F. Hatfull, David B. Hill, Miriam Braunstein

## ERRATUM

Volume 14, issue 3, e02655-25, 2025, https://doi.org/10.1128/spectrum.02655-25. “Phage Inactivation Buffer and Acidic pH”: “(PIB; 940 mM…” should read “(PIB; 40 mM…”

