# Supplementary material for: Inactivating conditions of therapeutic mycobacteriophages
Source: Microbiol Spectr. 2026 Feb 11;14(3):e02655-25. doi: 10.1128/spectrum.02655-25 (PMC12955380; doi:10.1128/spectrum.02655-25)
Supplement: Fig. S1 — M. smegmatis is not sensitive to phage inhibition buffer at pH 3. [file spectrum.02655-25-s0001.pdf]

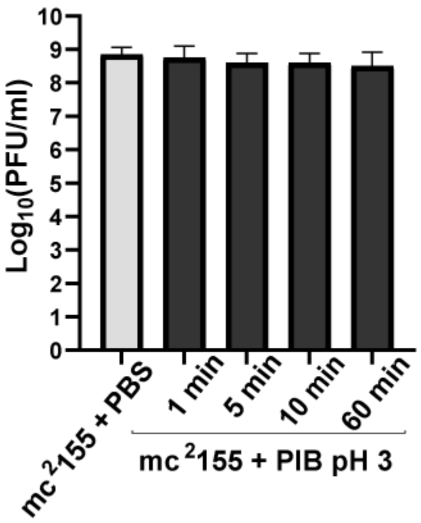

Supplemental Fig 1. *Mycobacterium smegmatis* is not sensitive to phage inhibition buffer (PIB) at pH 3. A saturated culture of *M. smegmatis* mc^{2}155 was pelleted, washed once in 1 X PBS, and resuspended in equal volume of 1 X PBS or in equal volume of PIB pH 3. The PIB pH 3 samples were incubated at room temperature incubation for up to 60-minutes and colony forming units (CFU) were quantified. Mean result from two independent experiments is plotted. No statistical significance was observed by one-way ANOVA ($p<0.05$) with Dunnett's post-test.
